# Supplementary material for: High proton conductivity within the ‘Norby gap’ by stabilizing a perovskite with disordered intrinsic oxygen vacancies
Source: Nat Commun. 2023 Nov 17;14:7466. doi: 10.1038/s41467-023-43122-4 (PMC10656576; doi:10.1038/s41467-023-43122-4)
Supplement: Supplementary file 1 — Supplementary Information [file 41467_2023_43122_MOESM1_ESM.pdf]

Supplementary information

High Proton Conductivity within the 'Norby gap' by Stabilizing a Perovskite with Disordered Intrinsic Oxygen Vacancies

Kei Saito et al.

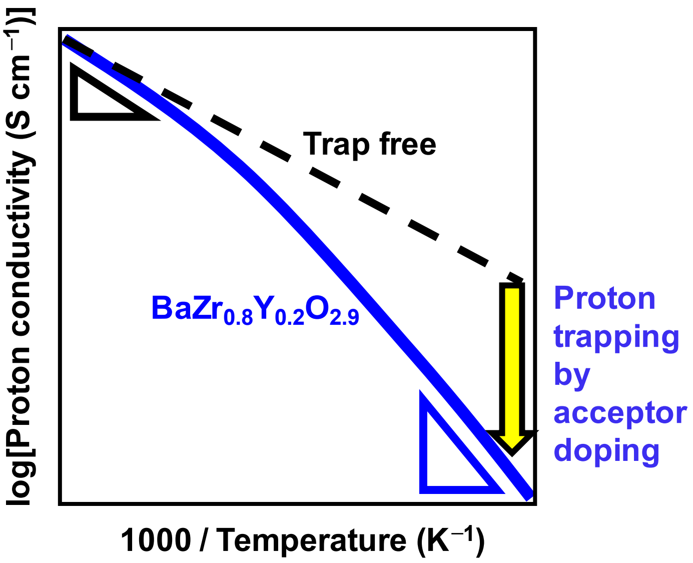

Supplementary Fig. 1. Schematic Arrhenius plot for proton conductivity of acceptor-doped $BaZrO_3$. In acceptor-doped $BaZrO_3$ such as $BaZr_{0.8}Y_{0.2}O_{2.9}$, the apparent activation energy for proton diffusion coefficient at intermediate and low temperatures is larger than that at high temperature due to the proton-dopant association energy, leading to high apparent activation energy for proton conductivity and low proton conductivity at intermediate and low temperatures (Supplementary Note no. 1)[1].

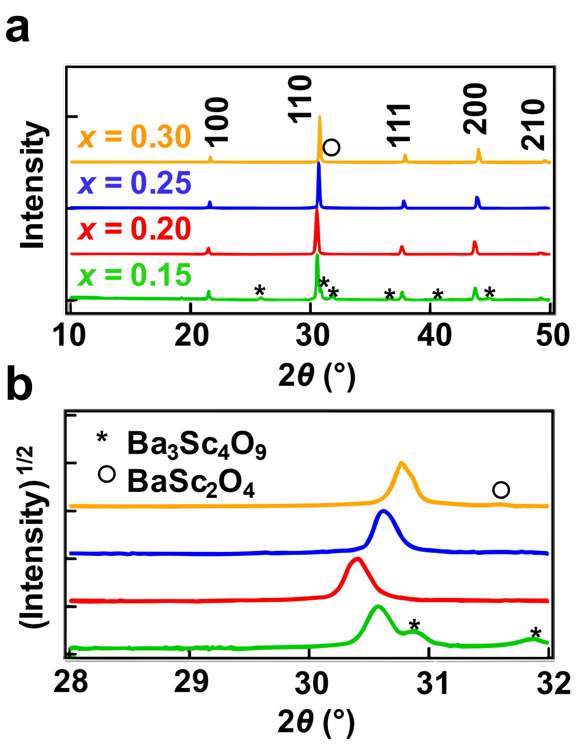

Supplementary Fig. 2. a,b Cu *K*α X-ray powder diffraction (XRD) patterns for the compositions of $x = 0.30$ (orange profile), 0.25 (blue profile), 0.20 (red profile) and 0.15 (green profile) in $BaSc_{1-x}Mo_xO_{2.5+3x/2-y/2}(OH)_y$ at 24 °C. Vertical axes of **a** and **b** are XRD intensity and square root of the XRD intensity, respectively. Asterisk and open circle denote the impurity phases $Ba_3Sc_4O_9$ and $BaSc_2O_4$, respectively. *hkl* denotes the reflection index of the primitive cubic cell. The refined lattice parameter of $x = 0.25$ sample (4.1266(17) Å) was smaller than that of $x = 0.20$ (4.1461(2) Å) at 24 °C. The smaller lattice parameter is mainly ascribed to the smaller ionic radius of Mo^{6+} (0.59 Å for coordination number of 6)[2] in comparison with that of Sc^{3+} (0.745 Å for coordination number of 6)[2], indicating the formation of solid solutions.

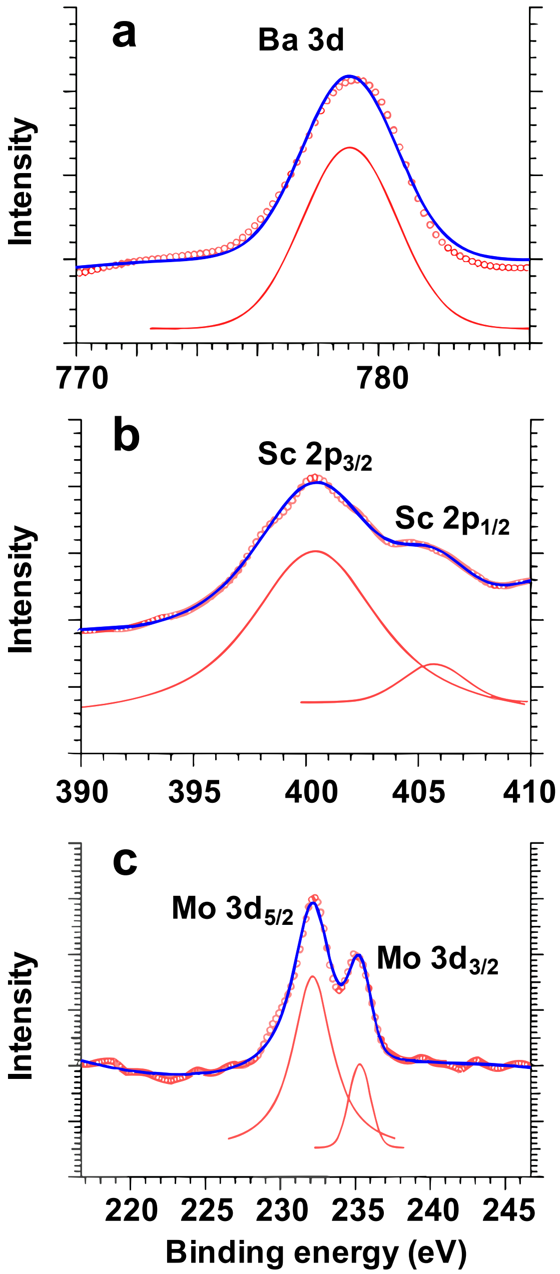

Supplementary Fig. 3. XPS specra of **a** Ba 3d, **b** Sc 2p, and **c** Mo 3d in $BaSc_{0.8}Mo_{0.2}O_{2.8-y/2}(OH)_y$ (BSM20) at 24 °C. The blue line and red open circles denote fitting line and experimental data, respectively. Red curve stands for each component obtained by the fitting. The obtained binding energies of Ba 3d (779.0 eV), Sc $2p_{3/2}$ (400.4 eV), Sc $2p_{1/2}$ (405.8 eV), Mo $3d_{5/2}$ (232.1 eV), and Mo $3d_{3/2}$ (235.2 eV) indicate Ba^{2+}, Sc^{3+}, and Mo^{6+} oxidation states, respectively[3,4,5].

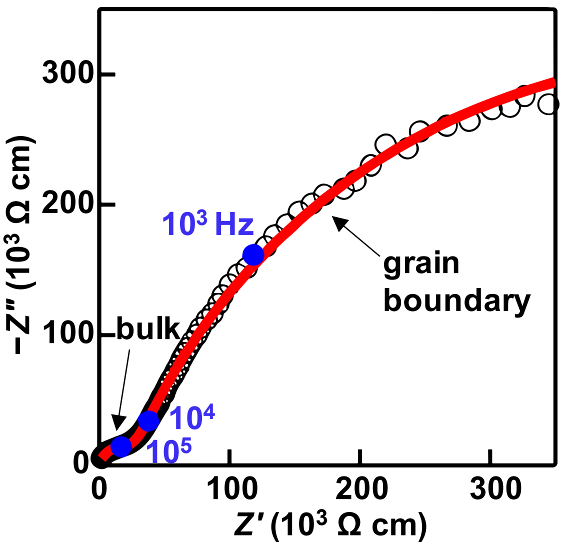

Supplementary Fig. 4. Complex impedance plots of BSM20 recorded in wet air at 70 °C. The number at each blue closed circle denotes the frequency. The red line represents the fitting curve, which indicates two semi-circles due to bulk and grain-boundary responses.

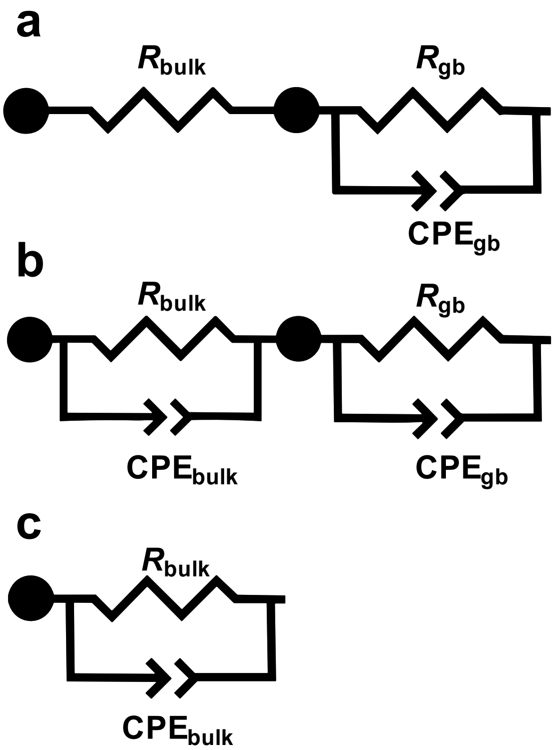

Supplementary Fig. 5. Equivalent circuits used to model the impedance spectra of BSM20 at **a** 525–93, **b** 70, and **c** 47 °C and $BaSc_{0.75}Mo_{0.25}O_{2.875-y/2}(OH)_y$ (BSM25) at **a** 485–178, **b** 130–87 and **c** 42 °C in wet air. *R*, *C*, and CPE denote a resistor, capacitance and constant phase element, respectively. The subscripts "bulk" and "gb" denote the bulk and grain boundary, respectively.

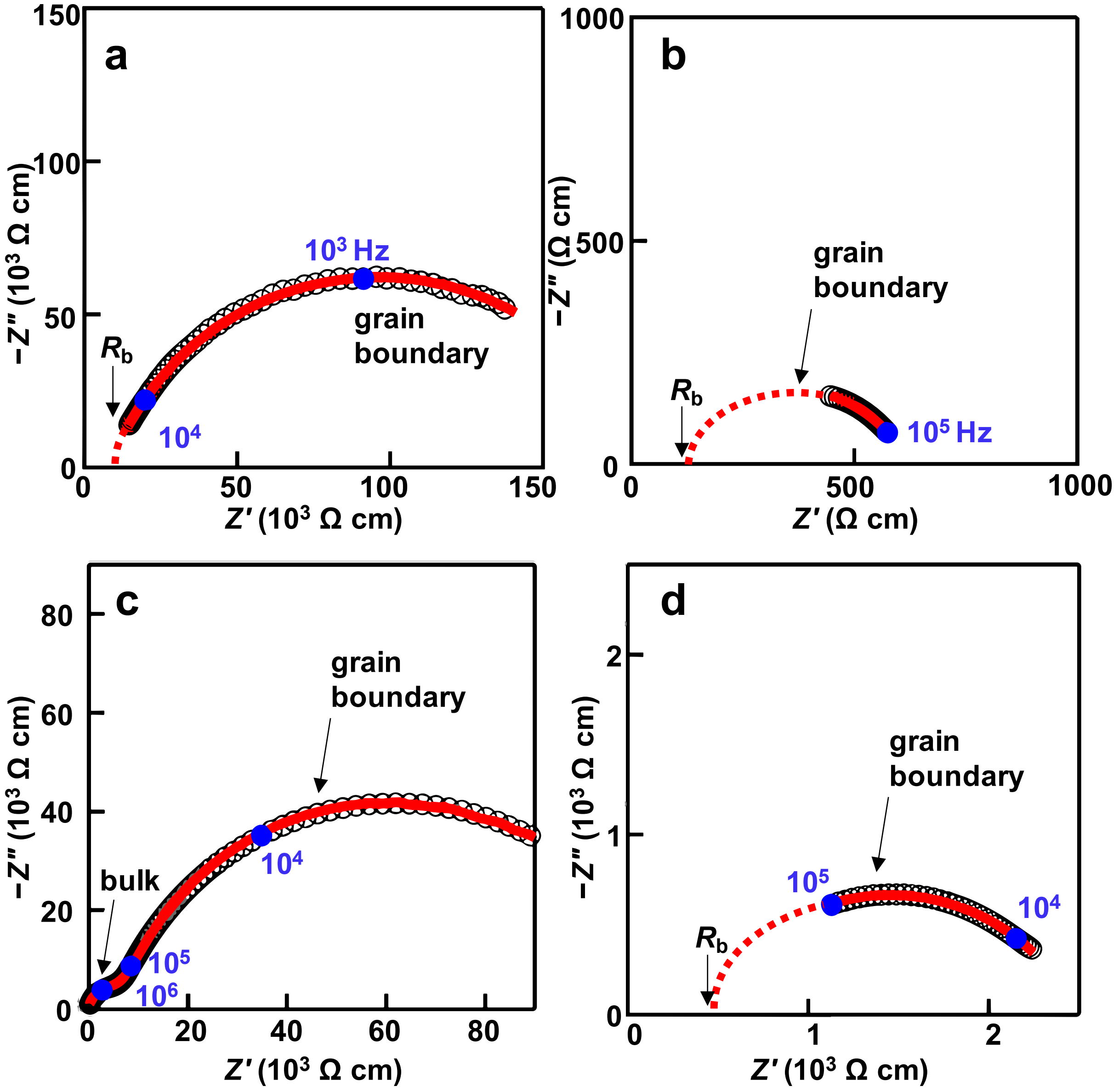

Supplementary Fig. 6. Complex impedance plots of BSM20 at **a** 93 and **b** 313 °C and BSM25 at **c** 130 and **d** 272 °C recorded in wet air. The number of each blue closed circle denotes the frequency. The red solid line represents the fitting curve, which indicates two semi-circles due to bulk and grain-boundary responses in panel **c** and a semi-circle due to grain-boundary response in panels **a**, **b**, and **d**. The red dotted line is the extrapolated fitting curve. R_b denotes the bulk resistance.

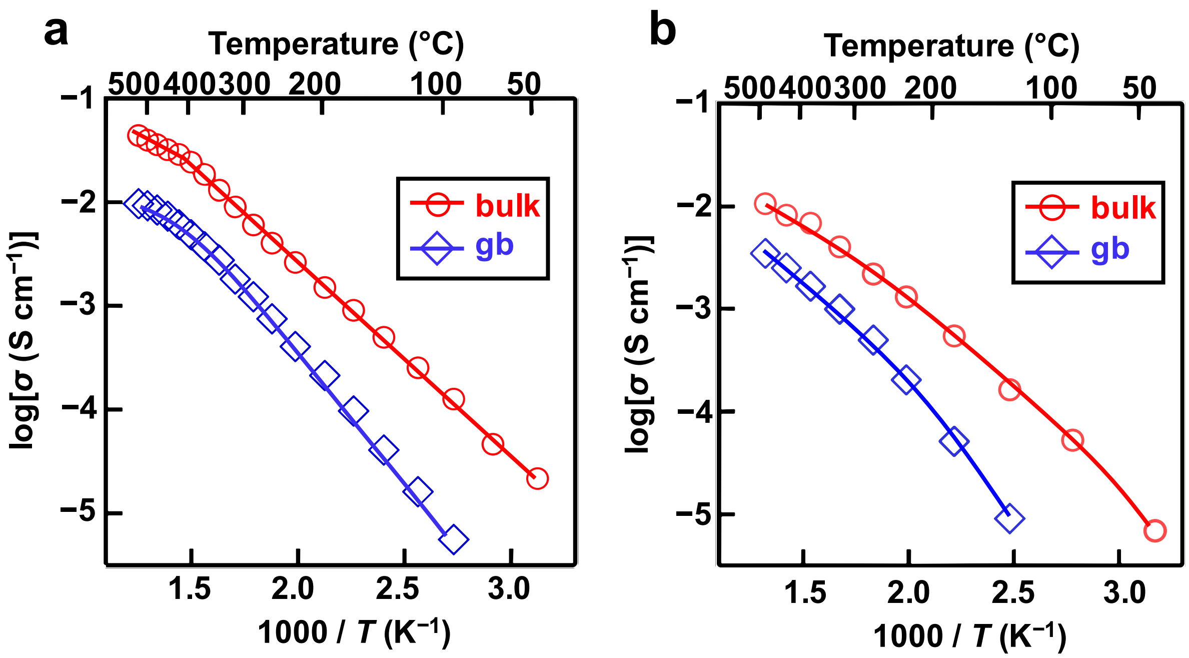

Supplementary Fig. 7. Arrhenius plots of bulk (red open circles) and grain-boundary (gb: blue open diamonds) conductivity in wet air of **a** BSM20 and **b** BSM25.

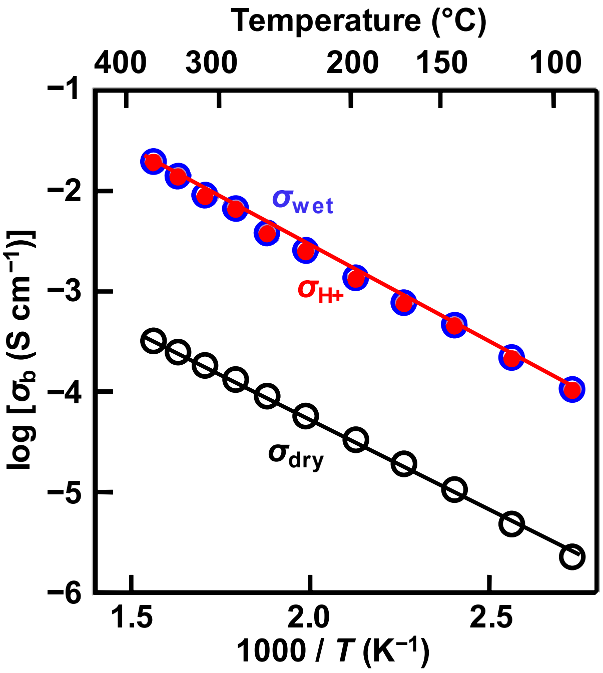

Supplementary Fig. 8. Arrhenius plots of bulk conductivity of BSM20 in wet N_2 gas (σ_{wet}, blue open circles) and dry N_2 gas (σ_{dry}, black open circles and line). Arrhenius plots of the bulk proton conductivity of BSM20 (red closed circles and line). Here, the bulk proton conductivity σ_{H+} was estimated using the equation, $\sigma_{H+} = \sigma_{wet} - \sigma_{dry}$. The proton transport number was calculated by the equation: $t_{H+} = \sigma_{H+}/\sigma_{wet}$. The obtained t_{H+} values were 98-100% between 93 and 367 °C.

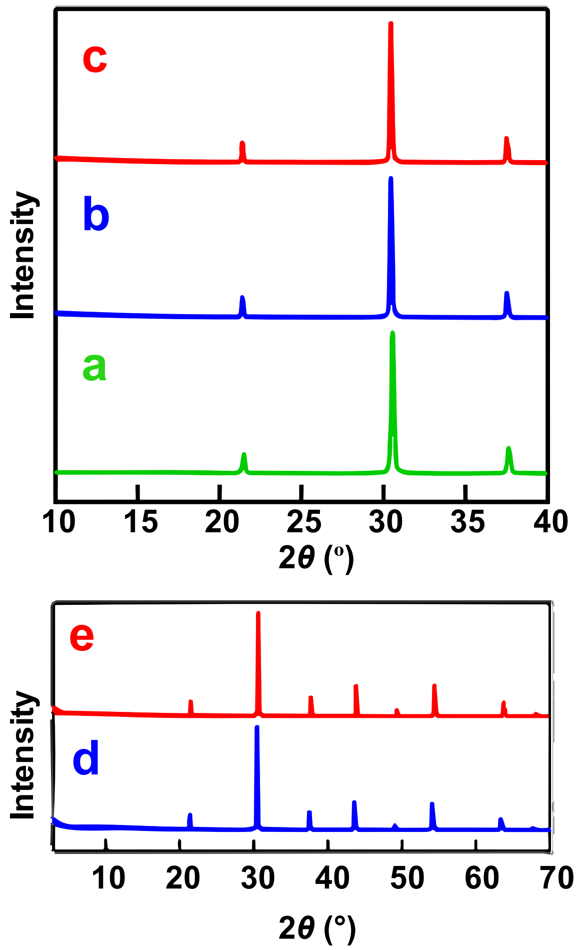

Supplementary Fig. 9. Cu *K*α XRD patterns of the powdered sample of BSM20 **a** before and after annealing in **b** O_2 and **c** 5% H_2 in N_2 at 320 °C, and those **d** before and **e** after annealing in CO_2 at 500 °C for 24 h. There were almost no differences between XRD patterns of the samples before and after annealing, which indicates the high chemical stability of BSM20.

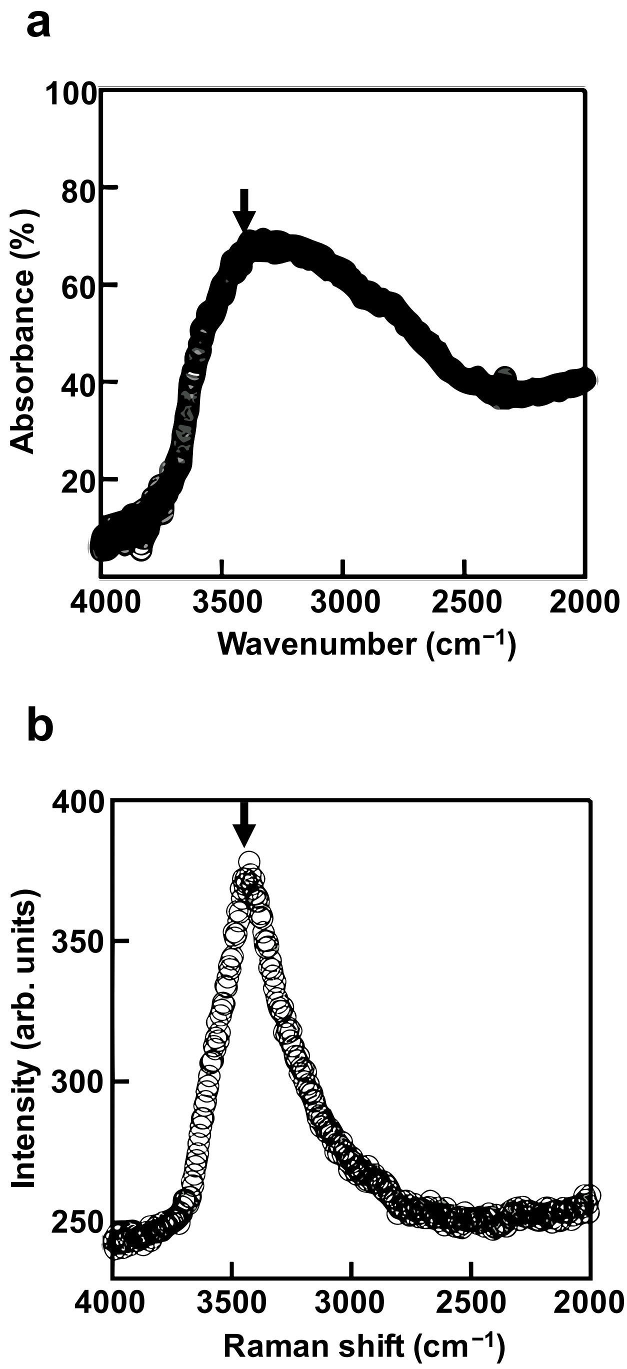

Supplementary Fig. 10. **a** IR and **b** Raman spectra of BSM20 sample. The arrow indicates the position of the OH bond. Novak proposed an empirical equation to express the correlation between OH bond length and frequency using data of 21 materials[6]. Using the empirical equation, the OH bond length of BSM20 was estimated to be 0.993 Å.

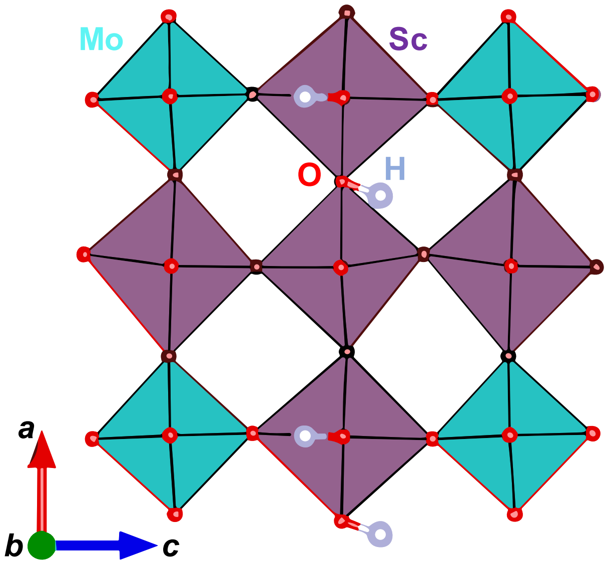

Supplementary Fig. 11. A part of the optimized structure of $Ba_8Sc_6Mo_2O_{23}(H_2O)$ (= $[BaSc_{0.75}Mo_{0.25}O_{2.875}\bullet 0.125\ H_2O]_8$ = $[BaSc_{0.75}Mo_{0.25}O_{2.75}\bullet 0.25\ (OH)]_8$), which was obtained by DFT calculations (Supplementary Table 3). Each H atom is coordinated to an oxygen atom of ScO_6 octahedron. It should be noted that an H atom is not coordinated to an oxygen atom of MoO_6 octahedron due to large electrostatic repulsion between Mo and H atoms.

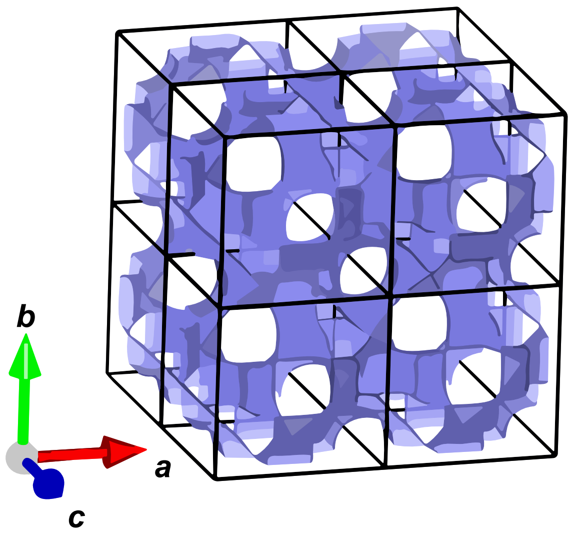

Supplementary Fig. 12. Isosurface of the bond-valence-based energy at 0.43 eV for a test proton of BSM20, which were calculated for the crystal parameters refined using the neutron diffraction data of BSM20 at 27 °C. Energy barrier for proton migration was estimated to be 0.41 eV, which agrees well with the experimental activation energy for bulk diffusion coefficient 0.4139(18) eV. Black cube denotes a unit cell.

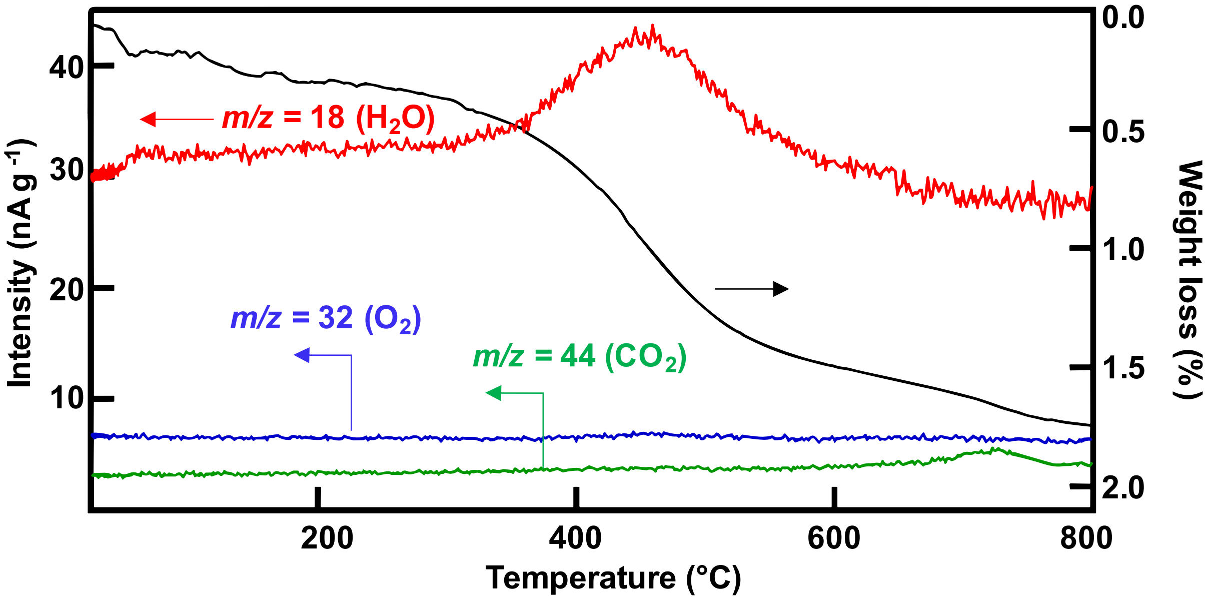

Supplementary Fig. 13. Thermogravimetric-mass spectrometric (TG-MS) data of BSM20 measured under dry He flow. The sample weight decreases during heating. MS measurements confirmed that the released gas is mainly H_2O molecules (*m/z* = 18, red line). Additional weight loss was observed between 700 and 800 °C due to the release of CO_2 gas (*m/z* = 44, green line). Significant O_2 molecules (*m/z* = 32, blue line) were not detected.

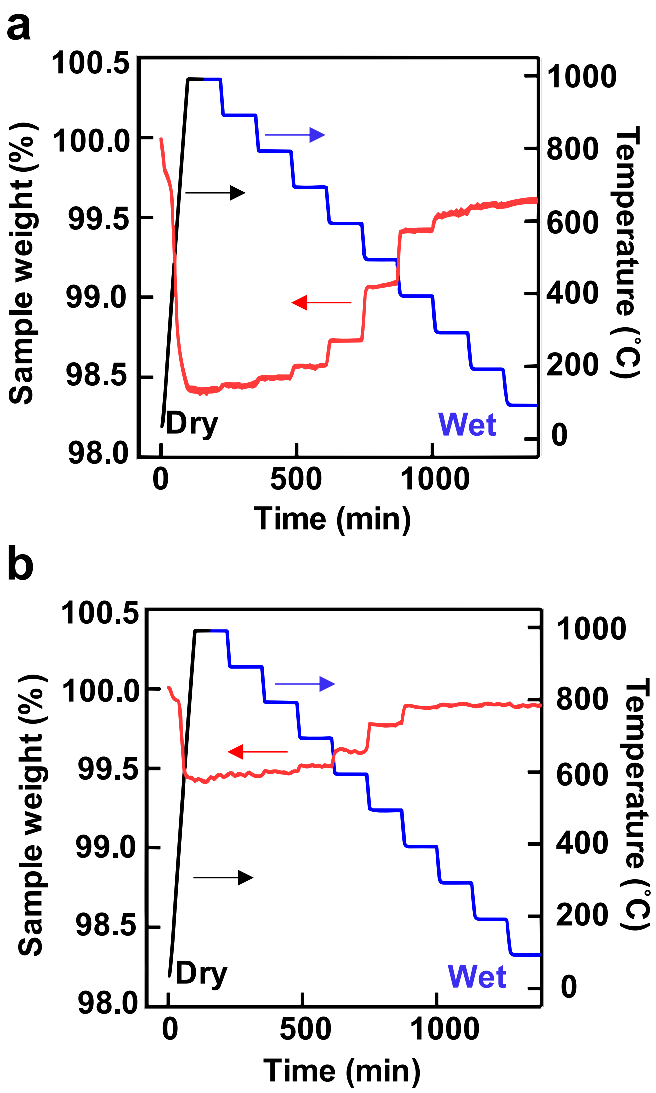

Supplementary Fig. 14. Water uptake of **a** BSM20 and **b** BSM25 in wet air. Using the data of water uptake, the hydration equilibrium constant, hydration enthalpy and hydration entropy were calculated (Supplementary Fig. 17, Supplementary Table 6, and Supplementary Note no. 3).

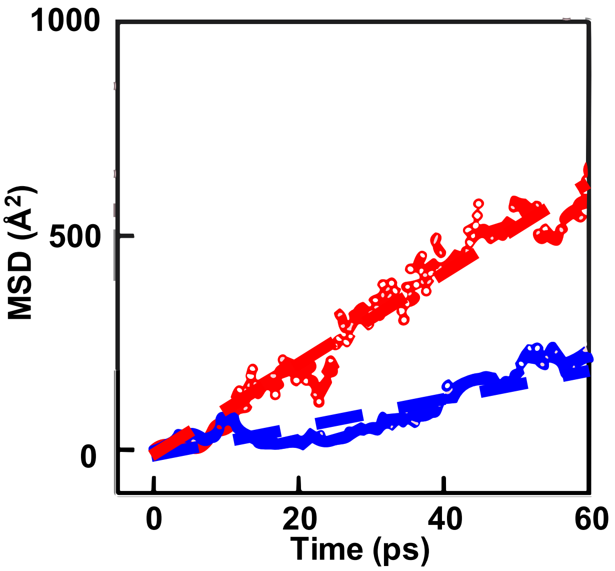

Supplementary Fig. 15. Mean square displacement (MSD) of protons in $Ba_8Sc_6Mo_2O_{23}(H_2O)$ obtained by *ab initio* molecular dynamics simulation at 1500 °C (red circles) and 700 °C (blue circles). The dashed line is a guide for eyes.

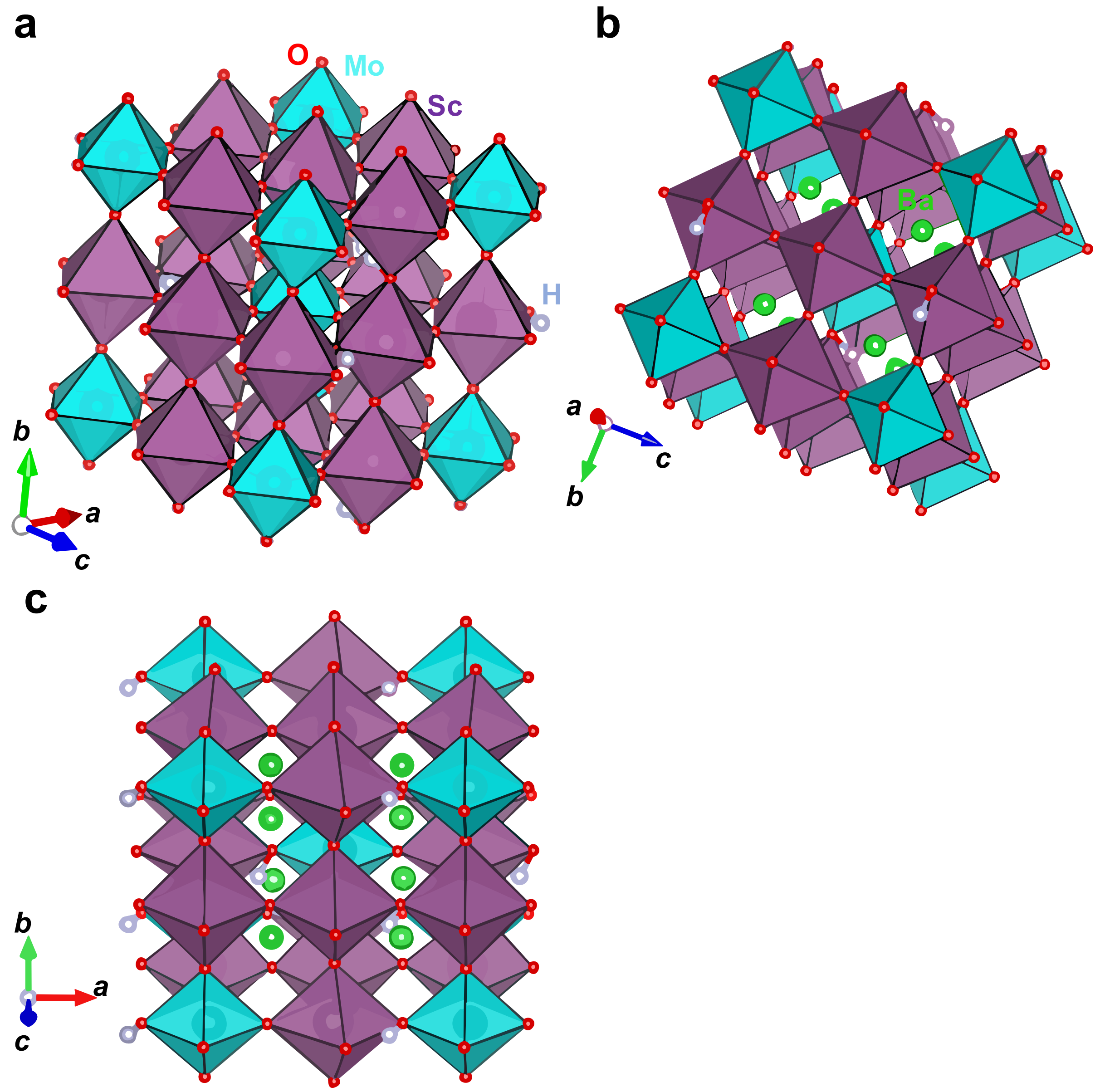

Supplementary Fig. 16. Optimized structures of $Ba_8Sc_6Mo_2O_{23}(H_2O)$ (= $[BaSc_{0.75}Mo_{0.25}O_{2.875}•0.125\ H_2O]_8$ = $[BaSc_{0.75}Mo_{0.25}O_{2.875}•0.25\ (OH)]_8$), which were obtained by static DFT calculations. **a** Each H atom is coordinated to an oxygen atom of ScO_6 octahedron. **b** One H atom is coordinated to an oxygen atom of ScO_6 octahedron and the other H atom is coordinated to an oxygen atom of MoO_6 octahedron. **c** Each H atom is coordinated to an oxygen atom of MoO_6 octahedron. The energy of model in **c** was 24 meV per atom higher than that of model shown in **b**. The energy for the model of **b** was 11 meV higher than that of **a**. These results support the electrostatic repulsion between the donor $Mo_{Sc}^{\cdot\cdot\cdot}$ and proton $H^{\cdot}$. The model of **a** has the minimum energy among the three models, therefore, the model of **a** was used as the initial structure in the AIMD simulations.

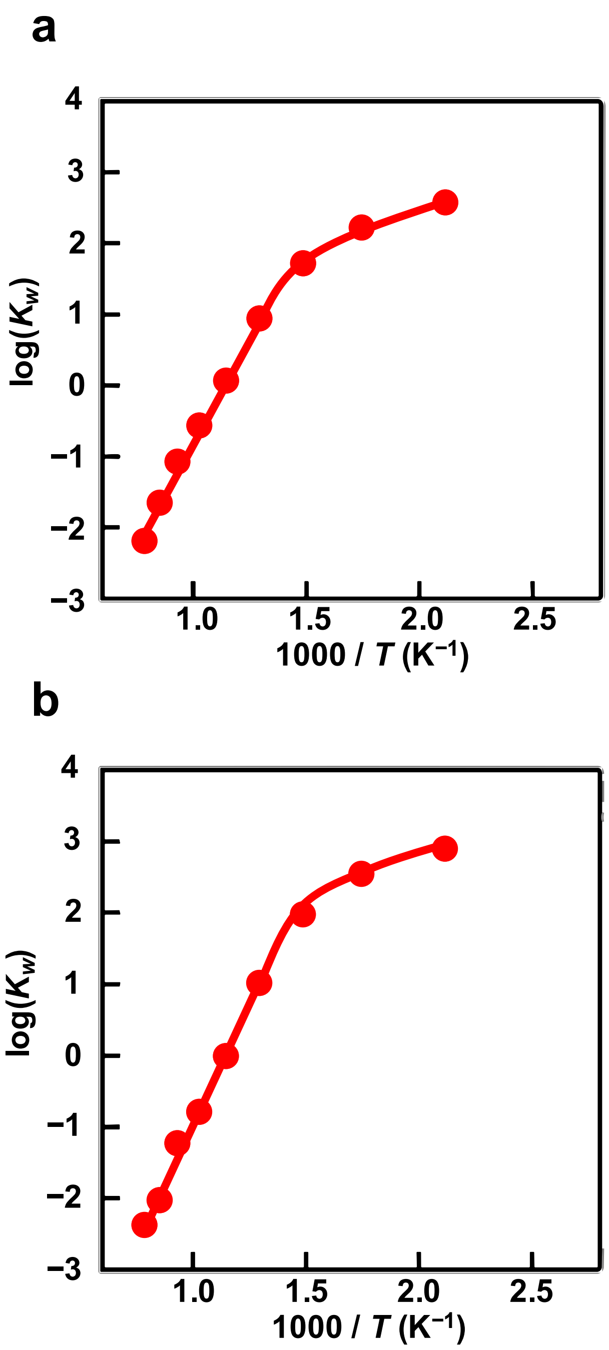

Supplementary Fig. 17. van 't Hoff plots of the equilibrium constant K_w for the hydration of **a** BSM20 and **b** BSM25. The details of the calculations of K_w values were described in the Supplementary Note no. 3.

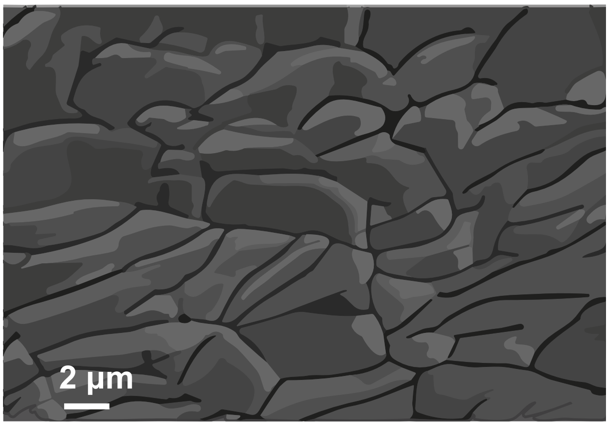

Supplementary Fig. 18. SEM micrograph of BSM20. The sample was polished and thermally etched at 1300 °C for 10 min prior to the SEM observation. The average grain size was estimated to be 2.2 μm in diameter by the intercept method.

Supplementary Table 1. Capacitances for bulk C_b and grain boundary C_{gb} of **a** BSM20 and **b** BSM25 in wet air.

a

T (°C)	C_b (F)	C_{gb} (F)
47	5.93×10^{-12}	
70	8.39×10^{-12}	2.87×10^{-10}
93		1.99×10^{-10}
117		1.80×10^{-10}
143		1.66×10^{-10}
169		1.48×10^{-10}
197		1.29×10^{-10}
230		1.08×10^{-10}
259		8.76×10^{-11}
285		7.08×10^{-11}
313		5.82×10^{-11}
340		4.10×10^{-11}
367		3.22×10^{-11}
394		2.77×10^{-11}
420		2.37×10^{-11}
447		2.14×10^{-11}
473		1.89×10^{-11}
499		1.70×10^{-11}
525		1.57×10^{-11}

b

T (°C)	C_b (F)	C_{gb} (F)
42	4.11×10^{-12}	
87	5.37×10^{-12}	5.45×10^{-11}
130	5.18×10^{-12}	4.81×10^{-11}
178		3.94×10^{-11}
229		2.91×10^{-11}
272		2.22×10^{-11}
324		1.48×10^{-11}
378		1.13×10^{-11}
431		1.12×10^{-11}
485		9.11×10^{-12}

Supplementary Table 2. H/D isotope effect of BSM20 on the activation energy E_a and pre-exponential factor A of BSM20. Here, E_D and A_D are the activation energy and pre-exponential factor of BSM20 in D_2O-saturated air, respectively. E_H and A_H are the activation energy and pre-exponential factor of BSM20 in H_2O-saturated air, respectively.

Atmosphere	E_a (eV)	A (K S cm^{-1})	$E_D - E_H$ (eV)	A_H/A_D
H_2O saturated air	0.41	1.7×10^4	0.04	0.59
D_2O saturated air	0.45	2.9×10^4		

Supplementary Table 3. Atomic coordinates of hydrogen atom of BSM20 (this work; Fig. 4d; Table 1), $BaTi_{0.3}Sc_{0.7}O_{2.65-y/2}(OD)_y$ (Ref. [7]) and $BaZr_{0.5}In_{0.5}O_{2.75-y/2}(OD)_y$ (Ref. [8]), which were refined by Rietveld analysis of neutron diffraction data. Atomic coordinates of proton of $Ba_8Sc_6Mo_2O_{23}(H_2O)$, which were optimized by DFT calculations (this work; Supplementary Fig. 11).

Composition	*x*	*y*	*z*
$BaSc_{0.8}Mo_{0.2}O_{2.6400(15)}(OD)_{0.3173(17)}$	0.4254(10)	0.2307(15)	0
$BaTi_{0.3}Sc_{0.7}O_{2.65-y/2}(OD)_y$	0.399(6)	0.241(8)	0
$BaZr_{0.5}In_{0.5}O_{2.75-y/2}(OD)_y$	0.437(3)	0.212(5)	0
$Ba_8Sc_6Mo_2O_{23}(H_2O)$	0.419	0.222	0.000

Supplementary Table 4. Activation energies for bulk proton diffusion coefficient of BSM20 (this work), BSM25 (this work), $BaZr_{0.8}Y_{0.2}O_{2.9-y/2}(OH)_y$ (Ref. [9,10]), $BaZr_{0.4}Sc_{0.6}O_{2.7-y/2}(OH)_y$ (Ref. [11]), $BaZr_{0.8}Sc_{0.2}O_{2.9-y/2}(OH)_y$ (Ref. [11]), and $BaCe_{0.9}Y_{0.1}O_{2.95-y/2}(OH)_y$ (Ref. [12]).* The activation energy of BSM25 (0.37 eV) was lower than that of BSM20 (0.41 eV), which suggests that the high donor Mo concentration x is more critical than high Sc concentration ($1-x$) for reducing proton trapping in $BaSc_{1-x}Mo_xO_{2.5+3x/2-y/2}(OH)_y$.

Composition	Activation energy E_a (eV)	Reference
$BaSc_{0.8}Mo_{0.2}O_{2.8-y/2}(OH)_y$	0.41	This work
$BaSc_{0.75}Mo_{0.25}O_{2.875-y/2}(OH)_y$	0.37	This work
$BaZr_{0.8}Y_{0.2}O_{2.9-y/2}(OH)_y$	0.53	9
$BaZr_{0.8}Y_{0.2}O_{2.9-y/2}(OH)_y$	0.48	10
$BaZr_{0.4}Sc_{0.6}O_{2.7-y/2}(OH)_y$	0.47	11
$BaZr_{0.8}Sc_{0.2}O_{2.9-y/2}(OH)_y$	0.50	11
$BaCe_{0.9}Y_{0.1}O_{2.95-y/2}(OH)_y$	0.54	12

* Activation energies for bulk diffusion coefficient of protons D obtained using Nernst-Einstein equation $D = \sigma_b RT/F^2C$ where R is gas constant, T is absolute temperature, F is Faraday constant, σ_b is the measured bulk conductivity in wet atmosphere, and C is the proton concentration estimated from TG measurements.

Supplementary Table 5. Ratios D_0(BSM20)/D_0(composition) and exp(BSM20)/exp(composition) for proton diffusion coefficients (composition = $BaZr_{0.8}Y_{0.2}O_{2.9-y/2}(OH)_y$ (BZY20), $BaZr_{0.4}Sc_{0.6}O_{2.7-y/2}(OH)_y$ (BZS60), $BaZr_{0.8}Sc_{0.2}O_{2.9-y/2}(OH)_y$ (BZS20), and $BaCe_{0.9}Y_{0.1}O_{2.95-y/2}(OH)_y$ (BCY10)) in the temperature ranges of 50−170 ºC and 200−400 ºC. Here, D_0(BSM20) and exp(BSM20) denote the pre-exponential factor D_0 for proton diffusion coefficient of BSM20 and exponential factor $\exp(-E_a/kT)$ of BSM20, respectively.

Composition	D_0(BSM20)/D_0(composition)		exp(BSM20)/exp(composition)			
BZY20	0.8 (50-170 ºC)	4 (200-400 ºC)	9 (50 ºC)	5 (170 ºC)	0.8 (200 ºC)	0.8 (400 ºC)
BZS60	2 (50-170 ºC)	6 (200-400 ºC)	9 (50 ºC)	5 (170 ºC)	1 (200 ºC)	1 (400 ºC)
BZS20	2 (50-170 ºC)	4 (200-400 ºC)	40 (50 ºC)	10 (170 ºC)	6 (200 ºC)	3 (400 ºC)
BCY10	0.2 (50-170 ºC)	1 (200-400 ºC)	110 (50 ºC)	30 (170 ºC)	3 (200 ºC)	2 (400 ºC)

Supplementary Table 6. Hydration enthalpy and entropy of BSM20, BSM25, $BaZr_{0.4}Sc_{0.6}O_{2.7-y/2}(OH)_y$ and $BaZr_{0.9}Sc_{0.1}O_{2.95-y/2}(OH)_y$ at 500-1000, 500-1000, 450-1000, and 500-900 °C, respectively. The hydration enthalpy and entropy of BSM20 and BSM25 were estimated using the van 't Hoff plots (Supplementary Fig. 17).

Composition	$\Delta H°$ (kJ mol^{-1})	$\Delta S°$ (J K^{-1} mol^{-1})
$BaSc_{0.8}Mo_{0.2}O_{2.8-y/2}(OH)_y$	−115(4)	−130(4)
$BaSc_{0.75}Mo_{0.25}O_{2.875-y/2}(OH)_y$	−127(5)	−145(5)
$BaZr_{0.4}Sc_{0.6}O_{2.7-y/2}(OH)_y$	−121(2)	−117(2)
$BaZr_{0.9}Sc_{0.1}O_{2.95-y/2}(OH)_y$	−119(5)	−125

The hydration enthalpies and entropies of BSM20 and BSM25 have similar values with those of conventional proton conducting perovskites $BaZr_{1-x}Sc_xO_{3-x/2-y/2}(OH)_y$ (x = 0.1 and 0.6)[10,11].

Supplementary Note no. 1.

(1) Hydration and proton-dopant association in acceptor-doped $A^{2+}B^{4+}O_3$

Here we describe the hydration and proton-dopant association in the perovskite-type $A^{2+}B^{4+}O_3$-based oxides where A^{2+} and B^{4+} are relatively larger and smaller cations, respectively. The general strategy to enhance the proton conductivity is the creation of oxygen vacancies $v_O^{\bullet\bullet}$ (= v in the text) by acceptor M^{3+} doping into $A^{2+}B^{4+}O_3$ perovskite where M^{3+} is an acceptor dopant cation with lower valence 3+ than 4+ of host B^{4+} cation, forming $AB_{1-x}M_xO_{3-x/2}(v_O^{\bullet\bullet})_{x/2}$ where x is the content of M acceptor dopant (e.g., A = Ba, B = Zr, and M = Y for $BaZr_{0.8}Y_{0.2}O_{2.9}$). Here $v_O^{\bullet\bullet}$ denotes the oxygen vacancy with effective positive charge of +2 through the Kröger-Vink notation. The defect equation of the acceptor doping can be expressed as follows:

$$M_2O_3 + 2B_B^{\times} + O_O^{\times} \longrightarrow 2M_B' + v_O^{\bullet\bullet} + 2BO_2 \qquad \text{Eq. (S1),}$$

Here, M_B' represents M^{3+} cation at the B^{4+} site with effective negative charge of −1. Since $AB_{1-x}M_xO_{3-x/2}(v_O^{\bullet\bullet})_{x/2}$ has oxygen vacancies $v_O^{\bullet\bullet}$, it can react with water vapor to fill partially the oxygen vacancies and to form hydroxide ions $(OH)_O^{\bullet}$:

$$H_2O + v_O^{\bullet\bullet} + O_O^{\times} \rightleftarrows 2(OH)_O^{\bullet} \qquad \text{Eq. (S2),}$$

forming $AB_{1-x}M_xO_{3-x/2-y/2}(OH)_y$ where y is proton concentration. However, the acceptor doping results in 'proton trapping' near dopant M_B' with effective negative charge of −1:

$$M_B' + (OH)_O^{\bullet} \rightleftarrows (M_B' \cdot (OH)_O^{\bullet})^{\times} \qquad \text{Eq. (S3)}$$

where $(M_B' \cdot (OH)_O^{\bullet})^{\times}$ is the proton-dopant association. The proton trapping leads to high apparent activation energy for proton conductivity and low proton conductivity at intermediate and low temperatures (Supplementary Fig. 1).

(2) Hydration of donor-doped $A^{2+}B^{3+}O_{2.5}$

Here we describe the hydration in the perovskite-type $A^{2+}B^{3+}O_{2.5}$-based oxides where A^{2+} and B^{3+} are relatively larger and smaller cations, respectively. We express the defect reactions using the notation after Norby[13].* In cubic $ABO_{2.5}$ perovskite, the occupancy factors of oxygen atoms and intrinsic oxygen vacancies are 5/6 and 1/6, respectively, at the anion site, thus, we refer the anion site as $\frac{5}{6}\mathrm{O}$. The intrinsic oxygen vacancy has an actual charge of 0 so that its effective charge is +5/3 at the anion site $\frac{5}{6}\mathrm{O}$. Therefore, the oxygen vacancy at the anion $\frac{5}{6}\mathrm{O}$ site is denoted as $v_{\frac{5}{6}\mathrm{O}}^{\frac{5}{3}\bullet}$. In the text, the intrinsic oxygen vacancy is denoted as □ for simplicity, but in this Supplementary Note no. 1 (2), it is denoted as $v_{\frac{5}{6}\mathrm{O}}^{\frac{5}{3}\bullet}$. These $ABO_{2.5}$ oxides have 'intrinsic oxygen vacancy' enabling donor M^{6+} doping forming $AB_{1-x}M_xO_{2.5+3x/2}$ (e.g., A = Ba, B = Sc, M = Mo);

$$2MO_3 + 2B_B^{\times} + 3v_{\frac{5}{6}\mathrm{O}}^{\frac{5}{3}\bullet} \longrightarrow 2M_B^{\bullet\bullet\bullet} + 3\mathrm{O}_{\frac{5}{6}\mathrm{O}}^{\frac{1}{3}\prime} + B_2O_3 \qquad \text{Eq. (S4).}$$

Thus, materials with intrinsic oxygen vacancies $v_{\frac{5}{6}\mathrm{O}}^{\frac{5}{3}\bullet}$ can react with water vapor to fill partially the oxygen vacancies and to form hydroxide ions $(\mathrm{OH})_{\frac{5}{6}\mathrm{O}}^{\frac{2}{3}\bullet}$ at the anion $\frac{5}{6}\mathrm{O}$ site:

$$\mathrm{H_2O} + v_{\frac{5}{6}\mathrm{O}}^{\frac{5}{3}\bullet} + \mathrm{O}_{\frac{5}{6}\mathrm{O}}^{\frac{1}{3}\prime} \rightleftarrows 2(\mathrm{OH})_{\frac{5}{6}\mathrm{O}}^{\frac{2}{3}\bullet} \qquad \text{Eq. (S5)}$$

forming $AB_{1-x}M_xO_{2.5+3x/2-y/2}(OH)_y$ (y: proton concentration). Proton trapping by donor dopant $M_B^{\bullet\bullet\bullet}$ does not occur due to the electrostatic repulsion between the positively charged hydroxide ion $(\mathrm{OH})_{\frac{5}{6}\mathrm{O}}^{\frac{2}{3}\bullet}$ and positively charged dopant $M_B^{\bullet\bullet\bullet}$. The apparent activation energy for proton diffusion coefficient in donor-doped $A^{2+}B^{3+}O_{2.5}$ (e.g., Mo-doped $BaScO_{2.5}$) would be lower than that in acceptor-doped $A^{2+}B^{4+}O_3$ (e.g., Y-doped $BaZrO_3$), leading to lower activation energy for proton conductivity and high proton conductivity at intermediate and low temperatures.

* Alternatively, the $A^{2+}B^{3+}O_{2.5}$ can also be regarded as M^{3+}-doped $A^{2+}B^{4+}O_3$ ($AB_{1-z}M_zO_{3-z/2}(v_\mathrm{O}^{\bullet\bullet})_{z/2}$; z = 1). As an example, we consider $BaScO_{2.5}$ (A = Ba, B = Zr, M = Sc). $BaScO_{2.5}$ can be regarded as Sc-doped $BaZrO_3$ ($BaZr_{1-z}Sc_zO_{3-z/2}(v_\mathrm{O}^{\bullet\bullet})_{z/2}$; z = 1). In this case, there are the following two problems. Firstly, since the Sc concentrations are very high in $BaScO_{2.5}$ and $BaSc_{0.8}Mo_{0.2}O_{2.8}$, rather than considering Sc as a defect (dopant), it might be better to consider $BaScO_{2.5}$ as a mother material. Second problem is the proton trapping by the dopant cation Sc^{3+} with effective negative charge of −1 (Sc_Zr') compared with host Zr^{4+} cation in $BaZr_{1-z}Sc_zO_{3-z/2}(v_\mathrm{O}^{\bullet\bullet})_{z/2}$. Protons can migrate between oxygen atoms of ScO_6 octahedra due to the high Sc concentration (Fig. 7b,c in the manuscript). Therefore, the proton trapping by the dopant cation Sc^{3+} might be invalid. In contrast, when we consider $BaScO_{2.5}$ as the mother material, the proton trapping by the Sc cation does not occur but the proton migrates between oxygen atoms of ScO_6 octahedra, which is consistent with the

results of AIMD simulations (Fig. 7b,c). Therefore, $BaScO_{2.5}$ is a better mother material compared with $BaZrO_3$. Therefore, we express the defect reactions in $BaScO_{2.5}$ with intrinsic oxygen vacancies using the notation after Norby[13].

Supplementary Note no. 2.

The diffusion coefficient D can be expressed using the pre-exponential factor D_0 and exponential factor $\exp(-E_a/kT)$ as follows:

$$D = D_0 \exp(-E_a/kT) \qquad \text{Eq. (S6)},$$

Here, E_a, k, and T are activation energy for bulk diffusion coefficient, Boltzmann constant, and absolute temperature, respectively. We calculated D_0 and $\exp(-E_a/kT)$ for D using the E_a and D values of BSM20, $BaZr_{0.8}Y_{0.2}O_{2.9-y/2}(OH)_y$ (BZY20), $BaZr_{0.4}Sc_{0.6}O_{2.7-y/2}(OH)_y$ (BZS60), $BaZr_{0.8}Sc_{0.2}O_{2.9-y/2}(OH)_y$ (BZS20), and $BaCe_{0.9}Y_{0.1}O_{2.95-y/2}(OH)_y$ (BCY10) in the temperature ranges of 50−170 °C and 200−400 °C. Supplementary Table 5 shows the ratios D_0(BSM20)/D_0(composition) and exp(BSM20)/exp(composition) for proton diffusion coefficients (composition = BZY20, BZS60, BZS20, and BCY10) in the temperature ranges of 50−170 °C and 200−400 °C. Here, D_0(BSM20) and exp(BSM20) denote the pre-exponential factor D_0 for proton diffusion coefficient of BSM20 and exponential factor $\exp(-E_a/kT)$ of BSM20, respectively.

In the temperature range of 50−170 °C, the D_0 of BSM20 was lower than D_0 of BZY20 [D_0(BSM20) = 0.8 D_0(BZY20)], while the exponential factors of BSM20 were 9 and 5 times higher than those of BZY20 at 50 and 170 °C, respectively, [exp(BSM20) = 9 exp(BZY20) at 50 °C and exp(BSM20) = 5 exp(BZY20) at 170 °C]. Thus, the higher proton diffusion coefficient of BSM20 compared to BZY20 at 50−170 °C is attributable to the higher exponential factor (lower activation energy) of BSM20 compared to BZY20 at 50−170 °C. In the temperature range of 200−400 °C, the D_0 of BSM20 was 4 times higher than D_0 of BZY20 [D_0(BSM20) = 4 D_0(BZY20)], while the exponential factors of BSM20 were lower than those of BZY20 at 200 and 400 °C, respectively [exp(BSM20) = 0.8 exp(BZY20) at 200 and 400 °C]. Thus, the higher proton diffusion coefficient of BSM20 compared to BZY20 at 200−400 °C is attributable to the higher pre-exponential factor D_0 of BSM20 compared to BZY20 at 200−400 °C.

In the temperature range of 50−170 °C, the D_0 of BSM20 was 2 times higher than D_0 of BZS60 [D_0(BSM20) = 2 D_0(BZS60)], while the exponential factors of BSM20 were 9 and 5 times higher than those of BZS60 at 50 and 170 °C, respectively, [exp(BSM20) = 9 exp(BZS60) at 50 °C and exp(BSM20) = 5 exp(BZS60) at 170 °C]. Thus, the higher proton diffusion coefficient of BSM20 compared to BZS60 at 50−170 °C is mainly attributable to the higher exponential factor (lower activation energy) of BSM20 compared to BZS60 at 50−170 °C. In the temperature range of 200−400 °C, the D_0 of BSM20 was 6 times higher than D_0 of BZS60 [D_0(BSM20) = 6 D_0(BZS60)], while the exponential factors of BSM20 were equal to those of BZS60 at 200 and 400 °C, respectively, [exp(BSM20) = exp(BZS60) at 200 °C and exp(BSM20) = exp(BZS60) at 400 °C]. Thus, the higher proton diffusion coefficient of BSM20 compared to BZS60 at 200−400 °C is attributable to the higher pre-exponential factor D_0 of BSM20 compared to BZS60 at 200−400 °C.

In the temperature range of 50−170 °C, the D_0 of BSM20 was 2 times higher than D_0 of BZS20 [D_0(BSM20) = 2 D_0(BZS20)], while the exponential factors of BSM20 were 40 and 10 times higher than those of BZS20

at 50 and 170 °C, respectively [exp(BSM20) = 40 exp(BZS20) at 50 °C and exp(BSM20) = 10 exp(BZS20) at 170 °C]. Thus, the higher proton diffusion coefficient of BSM20 compared to BZS20 at 50−170 °C is mainly attributable to the higher exponential factor (lower activation energy) of BSM20 compared to BZS20 at 50−170 °C. In the temperature range of 200−400 °C, the D_0 of BSM20 was 4 times higher than D_0 of BZS20 [D_0(BSM20) = 4 D_0(BZS20)], while the exponential factors of BSM20 were 6 and 3 times higher than those of BZS20 at 200 and 400 °C, respectively [exp(BSM20) = 6 exp(BZS20) at 200 °C and exp(BSM20) = 3 exp(BZS20) at 400 °C]. Thus, the higher proton diffusion coefficient of BSM20 compared to BZS20 at 200−400 °C is attributable to both the higher exponential factor (lower activation energy) and the higher pre-exponential factor D_0 of BSM20 compared to BZS20 at 200−400 °C.

In the temperature range of 50−170 °C, the D_0 of BSM20 was lower than D_0 of BCY10 [D_0(BSM20) = 0.2 D_0(BCY10)], while the exponential factors of BSM20 were 110 and 30 times higher than those of BCY10 at 50 and 170 °C, respectively [exp(BSM20) = 110 exp(BCY10) at 50 °C and exp(BSM20) = 30 exp(BCY10) at 170 °C]. Thus, the higher proton diffusion coefficient of BSM20 compared to BCY10 at 50−170 °C is attributable to the higher exponential factor (lower activation energy) of BSM20 compared to BCY10 at 50−170 °C.

In the temperature range of 200−400 °C, the D_0 of BSM20 was equal to D_0 of BCY10 [D_0(BSM20) = D_0(BCY10)], while the exponential factors of BSM20 were 3 and 2 times higher than those of BCY10 at 200 and 400 °C, respectively [exp(BSM20) = 3 exp(BCY10) at 200 °C and exp(BSM20) = 2 exp(BCY10) at 400 °C]. Thus, the higher proton diffusion coefficient of BSM20 compared to BCY10 at 200−400 °C is attributable to the higher exponential factor (lower activation energy) of BSM20 compared to BCY10 at 200−400 °C.

Supplementary Note no. 3.

Thermodynamic parameters for the hydration of BSM20 (x = 0.2 in $BaSc_{1-x}Mo_xO_{2.5+3x/2-y/2}(OH)_y v_{0.5-3x/2-y/2}$) and BSM25 ($x$ = 0.25) were obtained using active and inactive oxygen vacancies[11]. The hydration for the active oxygen vacancies can be expressed by the following equation.

$$H_2O + v(1)^{\frac{5}{3}\bullet}_{\frac{5}{6}O} + O^{\frac{1}{3}\prime}_{\frac{5}{6}O} \rightleftarrows 2(OH)^{\frac{2}{3}\bullet}_{\frac{5}{6}O} \qquad \text{Eq. (S7),}$$

Here, the $v(1)^{\frac{5}{3}\bullet}_{\frac{5}{6}O}$ is the active oxygen vacancy at the anion $\frac{5}{6}O$ site. Thus, the equilibrium constants K_w for the hydration [Eq. (S7)] can be expressed as:

$$K_w = \frac{[(OH)^{\frac{2}{3}\bullet}_{\frac{5}{6}O}]^2}{[v(1)^{\frac{5}{3}\bullet}_{\frac{5}{6}O}][O^{\frac{1}{3}\prime}_{\frac{5}{6}O}]P(H_2O)} \qquad \text{Eq. (S8)}$$

Here, $P(H_2O)$ is water vapor partial pressure. The total concentration of the active and inactive oxygen vacancies $[v^{\frac{5}{3}\bullet}_{\frac{5}{6}O}]$ is

$$[v^{\frac{5}{3}\bullet}_{\frac{5}{6}O}] = [v(1)^{\frac{5}{3}\bullet}_{\frac{5}{6}O}] + [v(2)^{\frac{5}{3}\bullet}_{\frac{5}{6}O}] \qquad \text{Eq. (S9)}$$

Here, $[v(2)^{\frac{5}{3}\bullet}_{\frac{5}{6}O}]$ is the concentration of the inactive oxygen vacancies. The sum of numbers of oxygen vacancies, oxide ions and hydroxide ions in $BaSc_{1-x}Mo_xO_{2.5+3x/2-y/2}(OH)_y v_{0.5-3x/2-y/2}$ equals to 3:

$$[v^{\frac{5}{3}\bullet}_{\frac{5}{6}O}] + [O^{\frac{1}{3}\prime}_{\frac{5}{6}O}] + [(OH)^{\frac{2}{3}\bullet}_{\frac{5}{6}O}] = 3 \qquad \text{Eq. (S10)}$$

Electroneutrality condition in $BaSc_{1-x}Mo_xO_{2.5+3x/2-y/2}(OH)_y v_{0.5-3x/2-y/2}$ can be expressed as follows assuming negligible hole concentration.

$$3[Mo^{\bullet\bullet\bullet}_{Sc}] + \frac{5}{3}[v^{\frac{5}{3}\bullet}_{\frac{5}{6}O}] + \frac{2}{3}[(OH)^{\frac{2}{3}\bullet}_{\frac{5}{6}O}] = \frac{1}{3}[O^{\frac{1}{3}\prime}_{\frac{5}{6}O}] \qquad \text{Eq. (S11)}$$

Substituting Eq. (S9) into Eq. (S10) yields

$$[v^{\frac{5}{3}\bullet}_{\frac{5}{6}O}] = \frac{1}{2}(1 - [(OH)^{\frac{2}{3}\bullet}_{\frac{5}{6}O}] - 3[Mo^{\bullet\bullet\bullet}_{Sc}]) \qquad \text{Eq. (S12)}$$

Substituting Eq. (S9), Eq. (S10) and Eq. (S12) into Eq. (S8) yields

$$K_w = \frac{4[(OH)^{\frac{2}{3}\bullet}_{\frac{5}{6}O}]^2}{(5-[(OH)^{\frac{2}{3}\bullet}_{\frac{5}{6}O}]+3[Mo^{\bullet\bullet\bullet}_{Sc}])(1-[(OH)^{\frac{2}{3}\bullet}_{\frac{5}{6}O}]-3[Mo^{\bullet\bullet\bullet}_{Sc}]-2[v(2)^{\frac{5}{3}\bullet}_{\frac{5}{6}O}])P(H_2O)} \qquad \text{Eq. (S13)}$$

The concentration of inactive oxygen vacancy $[v(2)^{\frac{5}{3}\bullet}_{\frac{5}{6}O}]$ was calculated by the following equation,

$$[v(2)^{\frac{5}{3}\bullet}_{\frac{5}{6}O}] = \frac{1}{2}(1 - 3[Mo^{\bullet\bullet\bullet}_{Sc}] - C_{H,Max}) \qquad \text{Eq. (S14)}$$

Here, $C_{\mathrm{H,Max}}$ is the measured maximum of proton concentration (i.e., proton concentration at 100 °C). The equilibrium constants K_{w} for van 't Hoff plots were calculated by Eq. (S13). The hydration enthalpy ΔH° and hydration entropy ΔS° were estimated by Eq. (S14) and van 't Hoff plots (Supplementary Fig. 17).

$$K_{\mathrm{w}} = \exp\left(-\frac{\Delta H^{\circ}}{RT}\right)\exp\left(\frac{\Delta S^{\circ}}{R}\right) \qquad \text{Eq. (S15)}$$

Here, R represents the gas constant.

Supplementary References

1. Yamazaki, Y. *et al.* Proton trapping in yttrium-doped barium zirconate. *Nat. Mater.* **12**, 647–651 (2013).
2. Shannon, R. D. & Prewitt, C. T. Effective ionic radii in oxides and fluorides. *Acta Crystallogr., Sect. B: Struct. Crystallogr. Cryst. Chem.***25**, 925–946 (1969).
3. Schmitz, P. J. Characterization of the surface of $BaCO_3$ powder by XPS characterization of the surface of $BaCO_3$ powder by XPS. *Surf. Sci. Spectra* **8**, 190–194 (2021).
4. Biesinger, M. C., Lau, L. W. M., Gerson, A. R., St, R. & Smart, C. Applied surface science resolving surface chemical states in XPS analysis of first row transition metals, oxides and hydroxides: Sc , Ti , V , Cu and Zn. *Appl. Surf. Sci.* **257**, 887–898 (2010).
5. Xiang, D., Han, C., Zhang, J. & Chen, W. Gap states assisted MoO_3 nanobelt photodetector with wide spectrum response. *Sci. Rep.* **4**, 4891 (2014).
6. Novak, A. Hydrogen bonding in solids correlation of spectroscopic and crystallographic data. in *Large Molecules*, Springer, Berlin, Heidelberg, 177–216 (1974).
7. Torino, N. *et al.* The influence of cation ordering, oxygen vacancy distribution and proton siting on observed properties in ceramic electrolytes: the case of scandium substituted barium titanate. *Dalton Trans.* **46**, 8387–8398 (2017).
8. Ahmed, I. *et al.* Location of deuteron sites in the proton conducting perovskite $BaZr_{0.50}In_{0.50}O_{3-y}$. *J. Alloys Compd.* **450**, 103–110 (2008).
9. Yamazaki, Y., Hernandez-Sanchez, R. & Haile, S. M. High total proton conductivity in large-grained yttrium-doped barium zirconate. *Chem. Mater.* **21**, 2755–2762 (2009).
10. Kreuer, K. D. *et al.* Proton conducting alkaline earth zirconates and titanates for high drain electrochemical applications. *Solid State Ion.* **145**, 295–306 (2001).
11. Hyodo, J., Kitabayashi, K., Hoshino, K., Okuyama, Y. & Yamazaki, Y. Fast and stable proton conduction in heavily scandium-doped polycrystalline barium zirconate at intermediate temperatures. *Adv. Energy Mater.* **10**, 2000213 (2020).
12. Kreuer, K. D. Aspects of the formation and mobility of protonic charge carriers and the stability of perovskite-type oxides. *Solid State Ion.* **125**, 285–302 (1999).
13. Norby, T. A Kröger-Vink compatible notation for defects in inherently defective sublattices. *J. Korean Ceram. Soc.* **47**, 19–25 (2010).
